# Modeling the leaf angle dynamics in rice plant

**DOI:** 10.1371/journal.pone.0171890

**Published:** 2017-02-16

**Authors:** Yonghui Zhang, Liang Tang, Xiaojun Liu, Leilei Liu, Weixing Cao, Yan Zhu

**Affiliations:** 1 Computer Engineering School, Weifang University, Weifang, P. R. China; 2 National Engineering and Technology Center for Information Agriculture, Jiangsu Key Laboratory for Information Agriculture, Jiangsu Collaborative Innovation Center for Modern Crop Production, Nanjing Agriculture University, Nanjing, P. R. China; Zhejiang University, CHINA

## Abstract

The leaf angle between stem and sheath (SSA) is an important rice morphological trait. The objective of this study was to develop and validate a dynamic SSA model under different nitrogen (N) rates for selected rice cultivars. The time-course data of SSA were collected in three years, and a dynamic SSA model was developed for different main stem leaf ranks under different N rates for two selected rice cultivars. SSA increased with tiller age. The SSA of the same leaf rank increased with increase in N rate. The maximum SSA increased with leaf rank from the first to the third leaf, then decreased from the third to the final leaf. The relationship between the maximum SSA and leaf rank on main stem could be described with a linear piecewise function. The change of SSA with thermal time (TT) was described by a logistic equation. A variety parameter (the maximum SSA of the 3^rd^ leaf on main stem) and a nitrogen factor were introduced to quantify the effect of cultivar and N rate on SSA. The model was validated against data collected from both pot and field experiments. The relative root mean square error (RRMSE) was 11.56% and 14.05%, respectively. The resulting models could be used for virtual rice plant modeling and plant-type design.

## Introduction

Rice is one of the most important food crops in the world. In the light of the constantly increasing demand for food, enhancing rice yield is always a challenging task for crop scientists. Much research has been conducted to achieve higher yield, and breeding for new rice plant types has become one of the most important methods [1, 2]. Virtual crop, through its integration of crop structural and functional processes, is becoming an important tool in breeding new crop plant types [3, 4], examples include wheat [5], maize [6–8], cotton [9], and other crops [10–13]. Leaf angle is a key trait for canopy structure, affecting canopy light distribution [14–16], and playing a crucial role in energy and mass balance in the soil-plant-atmosphere continuum [17].

Leaf angle distribution (LAD) affects the distribution of photosynthetic active radiation (PAR) on plant leaves, thus directly impacting plant productivity [15, 18]. Recently, many research results on modeling crop leaf angle have been reported. de Wit [19] proposed six special functions to simulate LAD characteristics in different crop canopy. Fuchs et al. used a Dual-parameter Beta function to describe LAD of different crop canopies [20]. Campbell employed an elliptic function to describe the probability density function of leaf angle [21], and this algorithm was improved with a rotating ellipsoid distribution function [22]. Verhoef used a linear combination of trigonometric functions to describe the probability density distribution of vegetation leaf angle [23]. A number of methods have been developed to measure leaf angles, for example, leveled digital photography [18]. Using simulations with the PROSAIL vegetation reflectance model and measurements of six field crops, two methods of determining leaf mean tilt angle (MTA) were proposed and evaluated, the central moment of LAD, from reflectance data in blue, red and near infrared wavebands [24]. Furthermore, crop leaf angle are affected by many factors, e.g. light condition [17], crop genotype, nitrogen level and plant density [5]. Leaf angle increased with increasing nitrogen rate [25]. Xu reported that reducing basal nitrogen resulted in improved canopy structure and increased the light transmittance [26].

**Fig 1:**
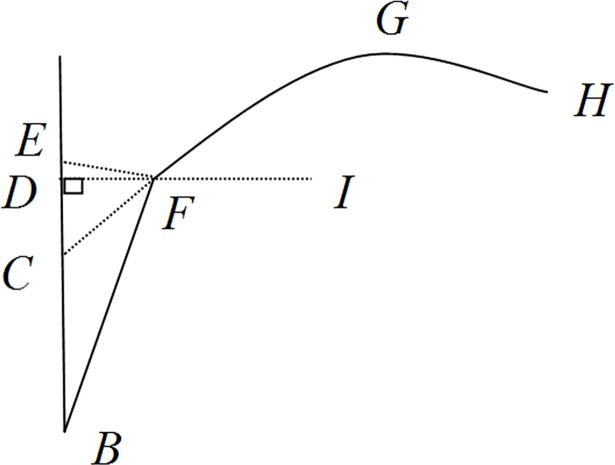
Schematic map of SSA.

As illustrated in Fig 1, there is a close relationship between SSA and leaf angle. Leaf angle (∠ECF) is equal to the sum of SSA (∠FBC) and the angle between leaf sheath and blade (SBA, ∠BFC), also called axial angle by Watanabe et al. [3]. Modeling SSA is an important component for rice plant-type design, PAR simulation and virtual rice. But existing studies mainly focus on LAD, data is lacking on SSA and on how SSA changes with N rate.

The objective of this study was to develop and evaluate a SSA model in relation to leaf rank under different N rates and for different cultivars. And this study mainly focused on modeling SSA of the isolated rice plant. The results would contribute to virtual plant modelling, rice plant-type design, and more accurate simulation of canopy light distribution.

## Material and methods

### Experiment site and design

Three experiments were conducted in 2010–2012 at the Experiment Station of Nanjing Agricultural University (118°50'E, 32°02'N), Jiangsu Province, China. For experiment 1, soil organic matter, total N, available phosphorous (P) and available potassium (K) were 14.5 g kg^−1^, 1.15 g kg^−1^, 41 mg kg^−1^, and 79 mg kg^-1^, respectively. Corresponding soil properties were 15.1g kg^−1^, 1.32g kg^−1^, 38 mg kg^−1^ and 85 mg kg^−1^, respectively for experiment 2, and were 13.3g kg^−1^, 1.41 g kg^−1^, 40 mg kg^−1^ and 87 mg kg^−1^, respectively for experiment 3.

Experiment 1: The experiment was conducted in 2010. Two rice cultivars, *Oryza sativa* L. ssp. japonica 'Wuxiangjing 14' with upright leaves (W14) and *Oryza sativa* L. ssp. indica 'Yangdao 6' with drooping leaves (YD6) were sown by direct seeding on 1 June. The culture pot is 35 cm in inside diameter at the top, 20 cm at the bottom, and 40 cm in height. Two seedlings were planted per pot for both cultivars. The treatments include three N rates of 0 g (N1), 1.5 g (N2), and 3.6 g (N3) each pot, which were applied in four splits (50% before planting, 10% at tillering, 20% at spikelet promotion, and 20% at spikelet protection stages). Ca_3_(PO_4_)_2_ and KCl were applied at a basal rate of 4.69 g and 2.88 g per pot, respectively. There were 40 pots for each cultivar and N rate combination. Other managements followed local practices for high yield in rice.

Experiment 2: The experiment was conducted in 2011. Both cultivars (YD6, W14) were grown under four N rates of 0 g (N1), 1.5 g (N2), 3.1 g (N3) and 4.6 g (N4) per pot. P_2_O_5_ and KCl were applied as a basal rate of 1.45 g and 2.88 g per pot, respectively. Other designs were the same as in experiment 1.

Experiment 3: The experiment was conducted in 2012. Two cultivars (YD6, W14) were planted on 26 May, and transplanted on 13 June. The plot size was 4 m×2.5 m with a plant spacing of 28 cm × 20 cm for YD6 and 20 cm × 15 cm for W14, with one seedling per hill for each cultivar. N rates were 0 kg ha^-1^(N1), 125 kg ha^-1^ (N2) and 250 kg ha^-1^(N3) with N applied in four splits (50% at pre-transplanting, 10% at tillering, 20% at spikelet promotion, and 20% at spikelet protection stages). P2O5 and K2O were applied at a basal rate of 82.5 kg ha^-1^and 164.7 kg ha^-1^, respectively. Other field managements followed local practices for high yield in rice.

### Data acquisition

Four rice plants with uniform growth status were tagged for each cultivar and N rate. During the tillering stage, the SSA of each leaf rank on main stem was measured by a protractor every other day. We repeated one measurement for five times, the average value of these five measured results was considered as the measured value of SSA, and the relative standard error (standard error/average value) of these five measured values was less than 10%. Six rice plants of each cultivar and N rate were destructively collected for samples at regular intervals. The growth status of the sampled plants was consistent with the tagged plants. During rice growing season, air temperature was recorded daily at 30-min intervals with the ZDR-11 (made at Zhejiang University of China; measuring range: -20°C~60°C; measuring accuracy: ±0.5°C; 3.6V lithium battery, the power under saving mode can be used continuously for more than 1 year; communication Interface: RS-232; sampling interval (optional): 2 seconds~24 hours). Data were downloaded every month and used for calculating thermal time (TT,°C·d) with a base temperature of 10°C for Japonica and 12°C for Indica cultivars [27]. In addition, N contents and dry weights of different organs from the sampled plants were measured by micro-Kjeldahl method [28] and an electronic scale, respectively.

### Date processing and analysis

Data from Experiment 2 were used to build the SSA model, data from experiment 1 and 3 were employed to validate the model. Data analysis were carried out with Microsoft Excel 2010, CurveExpert 1.4 and MatlabR2009a.

### The basic description of SSA dynamics on main stem

**Fig 2:**
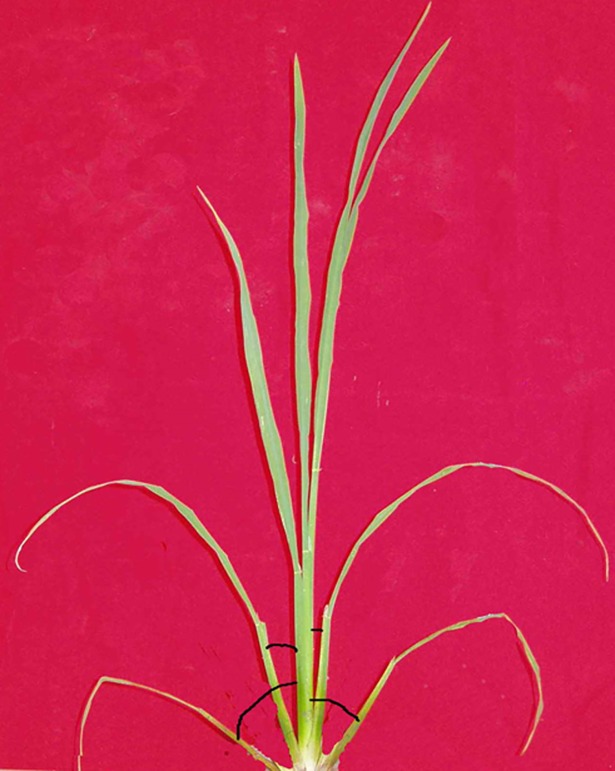
Real map of SSA.

In Fig 1, point *B* is the growing point of leaf sheath on main stem, *F* is the basal point of leaf blade. Point *E* is the position of *F* on main stem when the SSA (angle *EBF*) is *zero*, line *FC* is the tangent line. Angle *ECF* is the inclination angle of rice leaf blade, point *D* is the projection of *F* on main stem, and angle *BFG* is the angle between leaf sheath and leaf blade. The basal point of the leaf blade gradually offsets from the point *E* to *F* with the increasing of angle *FBE* (SSA), the offsetting can change the spatial distribution of rice leaf and canopy structure (Fig 2). Actually, the values of the angle *FBE* and *BFG* can be easily measured. Therefore angle *FCE* can be derived from the two angles as follows: ∠*FCE* = ∠*FBE*+(180^0^-∠*BFG*). The basal point *F* of leaf can be calculated by the angle *FBE* and the length of leaf sheath *BF*.

### SSA dynamics in relation to leaf rank and N rate

**Fig 3:**
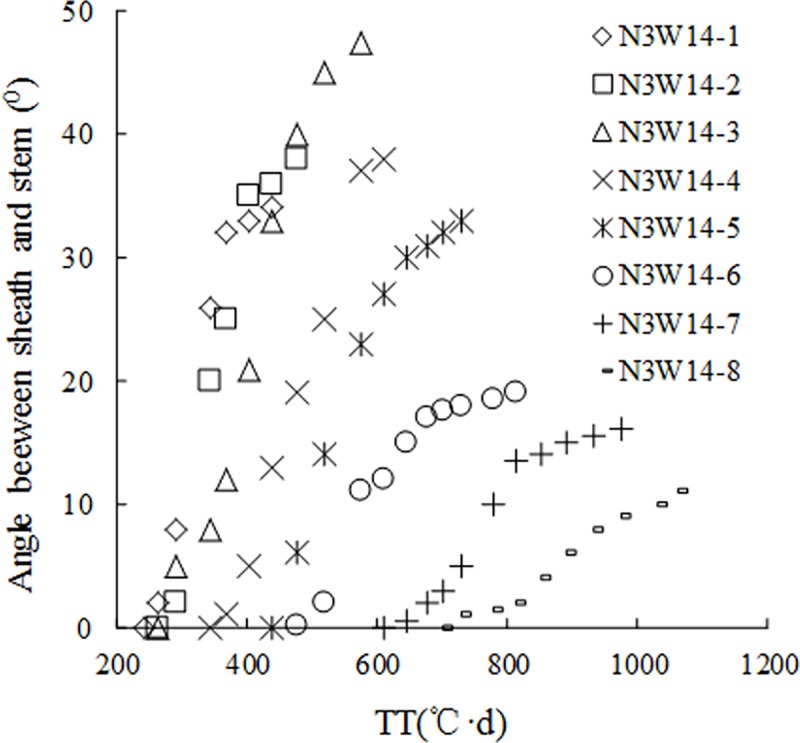
Changes of SSA in the 1^th^-8^th^ leaf ranks on main stem with TT in W14 under N3 rate.

**Fig 4:**
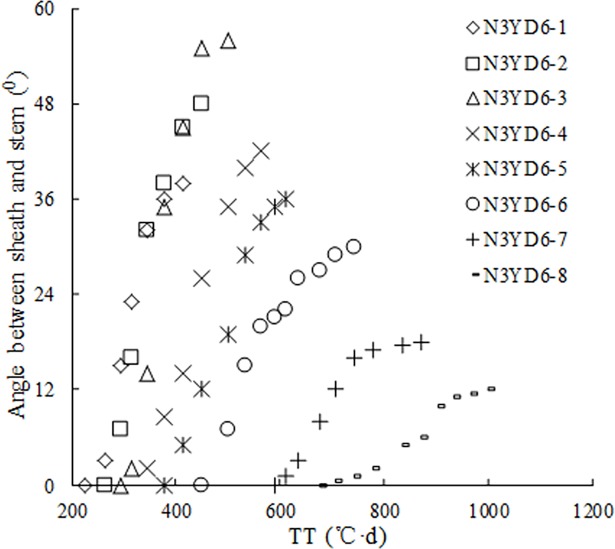
Changes of SSA in the 1^th^-8^th^ leaf ranks on main stem with TT in YD6 under N3 rate.

**Fig 5:**
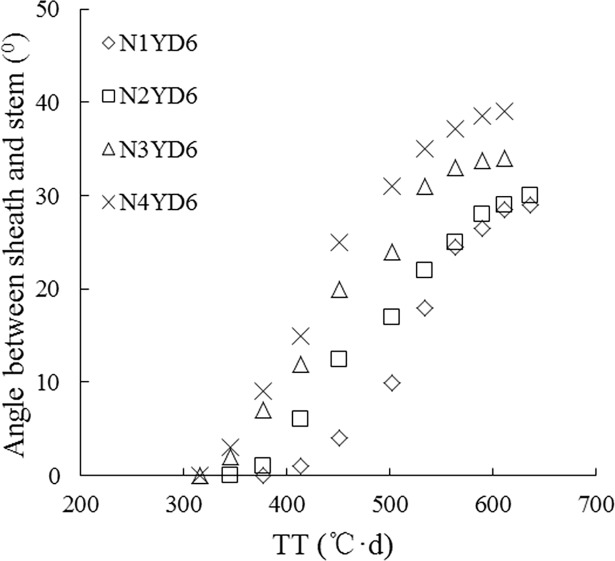
Changes of SSA in the 5^th^ leaf rank on main stem in YD6 under different N rates with TT.

**Fig 6:**
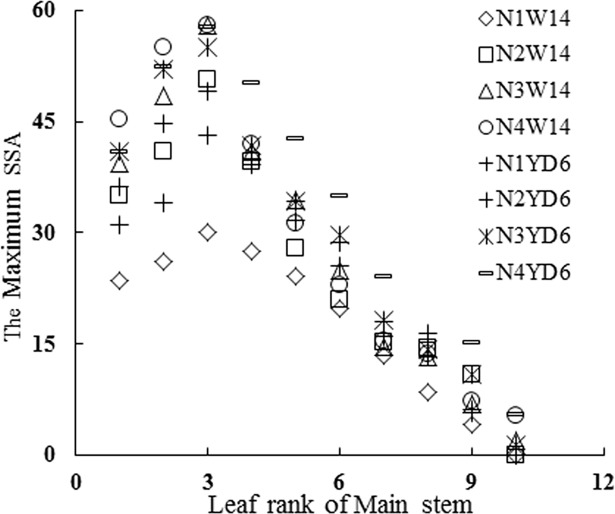
Changes of *MaxSSA*_n_ with leaf rank on main stem under different cultivars and N rates. Where, *MaxSSA*_n_ is the maximum value of SSA in leaf *n* on main stem.

**Fig 7:**
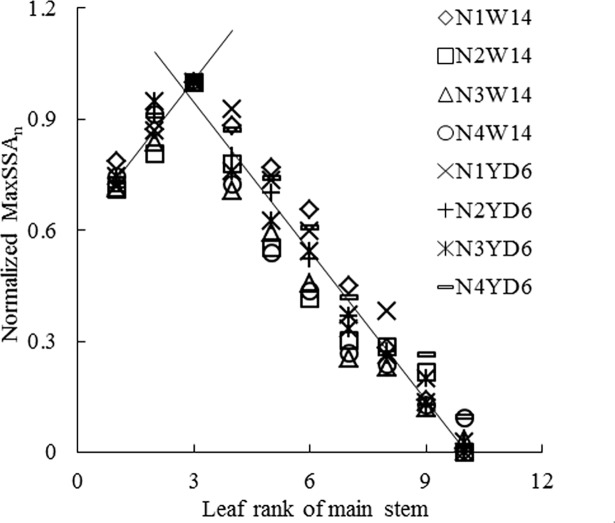
Changes of the normalized *MaxSSA*_n_ with leaf rank on main stem under different cultivars and N rates. Where, *MaxSSA*_n_ is the maximum value of SSA in leaf *n* on main stem.

Field observations indicated that SSA was *zero* before the first tiller appearance. The n^th^ tiller appeared with the emergence of the (*n+3*)^th^ leaf on main stem. SSA in leaf *n* gradually increased (Figs 1 and 2); SSA showed a more or less sigmoid pattern with thermal time (TT) (Figs 3 and 4); Although there were differences in SSA under different N rate for different leaf ranks, the same general pattern could be observed between different cultivars (Figs 3 and 4). As shown in Fig 5, SSA of the same leaf rank for a rice cultivar increased with increasing N rate. Maximum SSA on main stem first increased with leaf rank from the first to the third, and then decreased from the third to the final leaf, where the *MaxSSA*_3_ (the *MaxSSA*_n_ corresponding to the 3^rd^ leaf for each cultivar under different N rates) was maximum (Figs 6 and 7).

### The construction of SSA model

**Fig 8:**
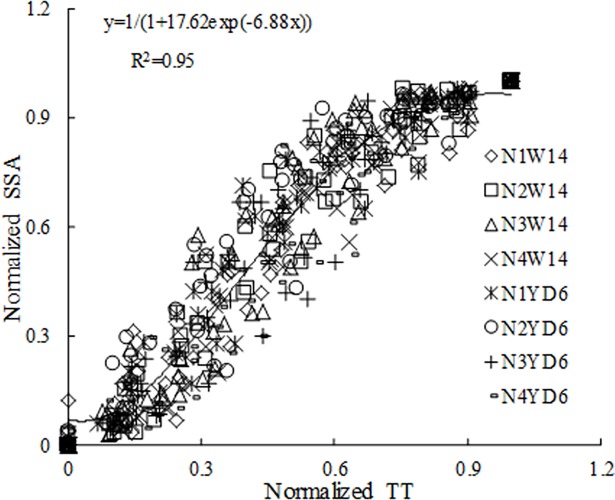
Changes of normalized SSA with normalized TT.

Measured SSA in each leaf rank under different cultivars and N rates were first normalized with regard to corresponding *MaxSSA*_*n*_ (Eq (1)). As shown in Fig 8, the normalized SSA demonstrated a sigmoid pattern in relation to normalized TT. Variance analysis showed no significant difference among different N rates and for different cultivars (P>0.05). Therefore, data were pooled to fit a uniform Logistic function (1)

$$\frac{SSA_{n}(TT)}{MaxSSA_{n}}=\left\{ \begin{array}{l} 0,\;,TT\leq ITT_{n};\\ \frac{1}{1+SSAa\times \exp(-SSAb\times NTT_{n})},\;ITT_{n}\leq TT\leq ITT_{n}+\Delta TT_{n};\\ 1,\;,ITT_{n}+\Delta TT_{n}\leq TT. \end{array}\right.$$ (1)

where the left side of Eq (1) is the normalized SSA and *TT* is the thermal time (°C). Experiment data showed that the relationship between the number of leaf appearance on main stem and TT accumulations after sowing could be described by a power function

$$ITT_{n}={\left(\frac{n+2}{a}\right)}^{\frac{1}{b}}$$ (2)

where *ITT*_n_ is the initial appearance time of SSA in leaf *n*, and is equal to the initial appearance time of leaf *n+3*. *ΔTT*_n_ is the lag in thermal time between the n^th^ and (n+3) ^th^ leaf age, calculated by Eq (3).

$$\Delta TT_{n}={\left(\frac{n+5}{a}\right)}^{\frac{1}{b}}-{\left(\frac{n+2}{a}\right)}^{\frac{1}{b}}$$ (3)

where *a* and *b* are equation coefficients, 0.0583 and 0.7936 (R^2^ = 0.9847) for YD6, and 0.0492 and 0.8062 (R^2^ = 0.9837) for W14.

The normalized *TT (NTT*_n_) was calculated by Eq (4)

$$NTT_{n}=\frac{TT-ITT_{n}}{\Delta TT_{n}}$$ (4)

The coefficients for *SSAa* and *SSAb* were 17.62 and 6.88 (R^2^ = 0.95), respectively; *SSA*_n_ (TT) is the value of SSA in leaf *n* at *TT* time. *MaxSSA*_n_ is the maximum value of SSA in leaf *n*.

There were 8 groups of *MaxSSA*_n_ (the maximum value of SSA in leaf *n*), corresponding to different cultivars and N rates, respectively. As shown in Fig 6, the *MaxSSA*_n_ first increased, then decreased with the leaf rank *n*. With maximum value occurred at the 3^rd^ leaf rank (*MaxSSA*_3_), and with similar patterns among these 8 groups. Since there were significant differences between groups (P<0.05), separate equations were developed for each data set. But once the data were normalized with regard to *MaxSSA*_3_ (Fig 7), there were no significant differences among the data sets (P>0.05). And data were fitted to a uniform piecewise linear function (5).

$$\frac{MaxSSA_{n}}{MaxSSA_{3}}=\left\{ \begin{array}{l} SLNa•n+SLNb,\;1\leq n\leq 3\\ SLNc•n+SLNd,\;4\leq n\leq 9 \end{array}\right.$$ (5)

where *SLNa* and *SLNb* are the coefficients of the first part in equation system, 0.1334 and 0.6054 (*R*^2^ = 0.9308), respectively; *SLNc* and *SLNd* are the coefficients of the second part in equation system, -0.1345 and 1.3507 (*R*^2^ = 0.9517), respectively. *MaxSSA*_3_ is the maximum value of SSA in the 3^rd^ leaf on main stem for each cultivar and N rate, calculated by Eq (6);

$$MaxSSA_{3}=MMaxSSA_{3}•FN$$ (6)

*MMaxSSA*_3_ is the maximum value of four *MaxSSA*_3_s for each cultivar under appropriate N rate, considered as a variety parameter, 58.0 and 50.0 for YD6 and W14 respectively, according to data analysis. *FN* is nitrogen impact factor, denoting the influence of nitrogen on *MMaxSSA*_3_, calculated by Eq (7);

$$FN=\left\{ \begin{array}{l} \frac{ANCSH}{MNCSH},ANCSH\leq MNCSH\\ 1\;,\;ANCSH\geq MNCSH \end{array}\right.$$ (7)

*ANCSH* is the actual nitrogen content of rice plant, measured by micro-Kjeldahl method [28]. *MNCSH* is the most appropriate nitrogen concentration of rice plant, calculated by Eq (8).

$$MNCSH=a•AGB^{-b}$$ (8)

where *a* and *b* are equation coefficients, 5.18 and 0.52, respectively. *AGB* is above-ground biomass of rice plant [27].

### Parameters calculation for validating SSA model

The measured SSAs of different leaf ranks under different cultivars and N rates in experiment 1 (2^nd^-6^th^ leaf) and 3 (3^rd^-6^th^ leaf) were used to validate the SSA model as follows: Firstly, *FN* was calculated by Eqs (7) and (8); Secondly, *MaxSSA*_3_ of two cultivars under three N rates was obtained by Eq (6), which were 46.0°, 51.8°and 56.3°for YD6, and 39.7°,44.2°and 45.8°for W14 in experiment 1 from the low to high N rate; while 47.0°, 52.86°and 55.1°for YD6, and 38.2°, 43.9°and 47.1°for W14 in experiment 3. Then, *MaxSSA*_n_ in each leaf (the 2^nd^ -6^th^ leaf for experiment 1, and the 3^rd^ -6^th^ leaf for experiment 3) for each cultivar and N rate were obtained by Eq (5); Finally, SSAs were simulated by Eq (1), with other parameters calculated with Eqs (2–4).

## Results

### The validation of SSA model

**Fig 9:**
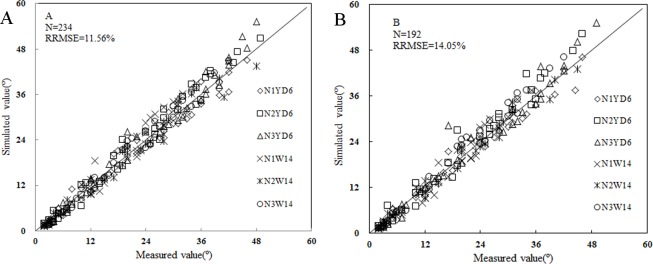
Comparisons of simulated and measured SSA under different cultivars and N rates.

The RRMSEs for experiment 1 were 11.17%、11.05% and 11.13% for YD6, 15.86%、10.46% and 10.17% for W14 for each N rate, respectively, with an overall RRMSE of 11.56% (Fig 9A); The RRMSEs for experiment 3 were 13.50%、14.32% and 13.87% for YD6, 14.65%、13.56% and 14.40% for W14, with an overall RRMSE of 14.05% (Fig 9B). The validation results showed good model performance in predicting the SSA dynamics in both pot and field experiments.

## Discussion

Light interception and utilization in crop canopy are important factors for crop production [29–31]. As shown in Fig 1, Leaf angle (∠ECF) is the sum of SSA (∠FBC) and the angle between leaf sheath and blade (SBA, ∠BFC), also called axial angle by Watanabe et al. [3]. This close relationship provides an indirect way for calculating leaf angle ECF with angles SSA and SBA. SSA affects leaf angle and therefore light distribution and light utilization of rice canopy, and is a key trait in rice morphology and canopy structure. But it was ignored or confused with leaf inclination angle in previous researches [4, 32]. Thus, some errors might have been introduced in simulating rice plant morphology and topology. Results from this study would provide improved simulations [3, 33].

The two rice cultivars involved in our experiments were of erect and droop leaf types. Originally, the data of *MaxSSA*_n_ (the maximum value of SSA in leaf *n*)(Fig 6) for two selected cultivars under different N rates could be simulated by 8 separate equations, but once a normalized method [34–35] was applied to process these data, there were no significant differences among the data sets (Fig 7). Meanwhile, the data of Fig 8 were also processed by this normalized method. The SSA of the same leaf rank on main stem for each cultivar increased with the increase of N rate (Fig 5). And the maximum value of each SSA of the same leaf rank increased with the increase of N rate (Fig 6). The similar effect of N rate on leaf angle with that on SSA has been found [25–26]. Besides, the tillering number per rice plant increased with the adding of N treatment. In our pot experiment of 2011, the average tillering number of single rice plant were 3.4, 4.7, 5.7, and 5.8 for W14 under N1, N2, N3, and N4, respectively. And corresponding number for YD6 were 3.7, 5.8, 6.8, and 7.8 under N1, N2, N3, and N4, respectively.

The growth and morphology of crop leaves are also affected by many factors, e.g. planting density [5], cultivar, water [8, 34], temperature, and N application. In our SSA model, there are three driving factors of cultivar, temperature (TT) and N application. Cultivar parameter of each rice cultivar is a constant. The interactions of temperature and N application mainly showed that the SSA growth rate demonstrated a sigmoid pattern with TT (Figs 3–5), the maximum value of SSA of each leaf rank increased with increasing N application (Fig 6). And the similar patterns have been investigated for leaf length and width of rice plant [35]. These factors affect leaf mass, leaf length and width [34], and thus leaf mass distribution along leaf axis. Besides, the rigidity and flexibility of blade and sheath (elasticity coefficient) also change, resulting in change in leaf curve [36–38].

Soil conditions can affect the growth of rice plant. Therefore, some differences exist in rice plant morphology between field and pot experiments. In our work, the SSA model was built by the data of experiment 2 (pot experiment). The validation values of SSA model for experiment 3 (field experiment) were correspondingly higher than those for experiment 1 (pot experiment). Besides, due to the changeable climate conditions of light intensity, wind, rain, and temperature, etc. there were also some differences among plant height, leaf length, and full heading day, etc. in our three experiments. Our SSA model would be adjusted and improved to more accurately estimate the leaf angle of rice plant under different planting modes and weather conditions.

The SSA model was developed only for main stem leaves in our work, but not tillers. Additional work is needed to determine whether tiller SSAs follows a pattern similar what is observed for main stems. In our experiments, the planting density was normal, rice plant could fully develop. Thus, the effect of planting density on SSA was not considered. In our later research, relevant density experiments would be conducted to help us for investigating the effect of planting density on SSA.

## Supporting information

S1 Table The data for figures in our study. (XLSX)
